# Bimanual coordination deficits in hands following stroke and their relationship with motor and functional performance

**DOI:** 10.1186/s12984-019-0570-4

**Published:** 2019-08-02

**Authors:** Chien-Hung Lai, Wen-Hsu Sung, Shang-Lin Chiang, Liang-Hsuan Lu, Chia-Huei Lin, Yi-Chun Tung, Chueh-Ho Lin

**Affiliations:** 1 Department of Physical Medicine and Rehabilitation, School of Medicine, College of Medicine, Taipei Medical University, 252 Wu-Hsing Street, Taipei, 11031 Taiwan, Republic of China; 2 Department of Physical Therapy and Assistive Technology, National Yang Ming University, 155 Linong Street, Sec. 2, Taipei, 112 Taiwan, Republic of China; 3 Department of Physical Medicine and Rehabilitation, Tri-Service General Hospital, School of Medicine, National Defense Medical Center, 325 Chenggong Road, Sec. 2, Neihu District, Taipei, 114 Taiwan, Republic of China; 4 School of Nursing & School of Medicine, National Defense Medical Center; Department of Nursing, Tri-Service General Hospital Songshan Branch, 325 Chenggong Road, Sec. 2, Neihu District, Taipei, 114 Taiwan, Republic of China; 5 Department of Physical Medicine and Rehabilitation, Taipei Medical University Hospital, 250 Wu-Xing Street, Taipei, 11031 Taiwan, Republic of China; 6 Master Program in Long-Term Care & School of Gerontology Health Management, College of Nursing, Taipei Medical University, 250 Wu-Xing Street, Taipei, 11031 Taiwan, Republic of China

**Keywords:** Coordination control, Movement disorder, Stroke, Grip force, Hands

## Abstract

**Background:**

Stroke can lead to movement disorders that affect interlimb coordination control of the bilateral upper extremities, especially the hands. However, few studies have investigated the influence of a stroke on bimanual force coordination control between the hands using a quantitative measurement tool, or the relationship of force coordination with paretic upper extremity motor and functional performance. We aimed to investigate these outcomes using a novel measurement device, and analyze the relationship of bimanual force coordination control deficits in both hands with motor and functional performances of the paretic upper extremity in stroke patients.

**Methods:**

Sixteen healthy adults and 22 stroke patients were enrolled. A novel bilateral hand grip measurement device with two embedded dynamometers was used to evaluate the grip force during a bilateral hand grip-force coordination control task. The alternating time and force applied for coordination with the grip force of both hands were calculated to analyze control of bimanual grip force coordination. Motor and functional measurements included the upper-extremity portion of the Fugl-Meyer assessment (FMA-UE), Wolf Motor Function Test (WMFT), Motor Assessment Scale (MAS), and Barthel Index (BI).

**Results:**

Compared with the healthy group, the alternating time from the non-paretic to the paretic hand was 27.6% shorter for stroke patients (*p* < 0.001). The grip force generated for coordination in the healthy group was significantly greater (30–59%) than that of the stroke group (*p* < 0.05), and the coefficients of variation of alternating time (*p* = 0.001) and force applied (*p* = 0.002) were significantly higher in the stroke group than the healthy group. The alternating time from the paretic to the non-paretic hand showed moderately significant correlations with the FMA-UE (*r* = − 0.533; *p* = 0.011), the WMFT (*r* = − 0.450; *p* = 0.036), and the BI (*r* = − 0.497; *p* = 0.019).

**Conclusions:**

Stroke results in a decline in bimanual grip force generation and increases the alternating time for coordinating the two hands. A shorter alternating time is moderately to highly associated with enhanced motor and functional performances.

## Background

Bilateral hand cooperation control is an essential function of the upper extremities and plays an important role in the activities of daily living (ADLs) [1, 2]. Additionally, interlimb coordination is an important function because it enables the individual to hold objects with two hands and exchange between the two hands to prevent objects from falling or slipping from the hands [3–5]. However, stroke results in muscle weakness, paresis, and spasticity of the paretic upper extremity. This can lead to disruption of interlimb coordination control, and increased dependency in ADLs due to motor impairment of the unilateral paretic limb and decreased cooperative movement of both hands [6–9]. Previous studies have shown that full recovery of the functional performance of the paretic upper extremity occurs in less than one-fifth of stroke patients [10–12], which means that many stroke patients will have a long-term movement disorder of cooperative control of the bilateral hands. Therefore, many studies have investigated the utility of bilateral movement training programs to enhance the motor and functional performances of the paretic upper extremity [8, 13, 14]. Appropriate tools for the direct measurement of coordination control of the grip force in both hands are important in the clinic, and could allow assessment of the functional status of bilateral hand cooperation control and enable the development of appropriate interventions to improve coordination deficits in both hands.

Clinical tests that are used to evaluate bilateral coordination control in stroke patients typically include the Functional Dexterity Test (FDT), the Minnesota Rate of Manipulation Test, and the Purdue Pegboard Manual Test [15–18]. However, these tests measure the dexterity of hand function by calculating the movement time and repetitions required to complete tasks using both hands simultaneously, and so they cannot directly determine changes in the grip force in coordination control between the two hands while simultaneously executing the tasks with both hands [15–19]. Combined electronic biosensors and computers have been developed in recent years and can be used to quantitatively analyze the coordination control among limbs. For example, Mose et al. developed a measurement system to demonstrate the impacts of aging on interactions between the bilateral hemispheres [20], and Lin et al. designed an assessment system with two embedded dynamometers to identify the influence of aging on the maximum grip-force output and capacity of coordination control of the hands [21]. Additionally, Kang and Cauraugh used an uncontrolled manifold (UCM) analysis to understand bilateral synergies (bilateral isometric force control) at three submaximal force levels for chronic stroke patients, which showed changes in bilateral synergies during isometric force control for those patients at different target force levels [22]. However, temporal changes in the grip-strength performance of both hands during coordination control tasks have rarely been discussed or demonstrated in stroke patients [21, 23]. Furthermore, few studies have investigated the relationship between the time and grip-strength performance of both hands during coordination control and the motor and functional performances of the paretic upper limb. The purposes of this study were, therefore, to investigate stroke-related changes in coordination control of grip force using two hands, and to analyze the relationship between coordination control of the hands and the motor and functional performances of the paretic upper extremity in stroke patients.

## Methods

### Participants

Sixteen healthy adults (23.4 ± 3.4 y/o) and 22 chronic stroke patients (53.7 ± 9.8 y/o) were invited to participate in this study. The inclusion criteria for healthy adults were the absence of disease that would affect the performance of upper limb movements and hand grip force generation. For the chronic stroke group, inclusion criteria were as follows: (1) the stroke event had occurred at least 6 months previously and cardiovascular condition was stable; (2) a unilateral ischemic or hemorrhagic stroke had occurred, as confirmed by collecting each patient’s medical history; (3) the patient was classified as Brunnstrom stage 3 or higher; (4) the patient had a Modified Ashworth Score of ≤3 for the wrist and finger joints and was able to flex and extend the paretic hand to hold a dynamometer [24]; (5) no other orthopedic or neurologic disorders existed; (6) the patient had a Mini-Mental State Examination score of ≥24 [14]. Exclusion criteria included feeling pain or discomfort during tasks [14]. Each participant signed an informed consent form before the study. This study was approved by the Joint Institutional Review Board of Taipei Medical University (no. N201605055). The demographic characteristics and clinical motor and functional outcome measurements for the stroke group are shown in Table 1.

**Table 1:** Demographic data and characteristics of the stroke patients

Gender	Age (years)	BH (m)	BW (kg)	Lesion area	On-set time (m)	Type	Lesion side	Brunnstrom stage	Modified ashworth scale	FMA-UE	BI	WMFT	MAS	MDT
F	48	1.62	60.8	Basal ganglia	15	Hemorrhage	L’t	4	1	11	55	28	1	NA
M	58	1.81	72	MCA	16	Infarction	R’t	6	0	57	55	60	18	4 min 59 s
F	63	1.48	47	MCA	19	Hemorrhage	L’t	5	2	13	60	19	1	NA
M	57	1.68	64	Basal ganglia	44	Hemorrhage	L’t	5	3	20	100	27	6	NA
M	60	1.72	69	Putamen	66	Hemorrhage	R’t	4	1	65	100	75	17	NA
M	67	1.65	69	Basilar artery and pontine	10	Infarction	R’t	6	1	60	95	75	16	NA
M	34	1.83	79	Left lentiform nucleolus	18	Hemorrhage	R’t	4	1	26	70	26	3	NA
M	60	1.72	79	Paramidian pons	14	Infarction	R’t	4	2	55	100	64	17	2 min 57 s
M	57	1.65	67	Bilateral parietal lobe	57	Infarction	R’t	4	3	35	60	45	11	NA
M	28	1.71	55	Frontotemporal region	13	Hemorrhage	L’t	4	1	27	70	32	4	NA
M	46	1.68	80	Basal ganglia	8	Hemorrhage	R’t	6	0	66	90	74	18	1 min 39 s
M	60	1.70	61	MCA	20	Infarction	L’t	6	1	54	95	56	14	5 min 6 s
M	47	1.72	70	Basal ganglia	67	Hemorrhage	R’t	5	3	47	80	53	14	7 min 15 s
F	57	1.54	57	M1 and M2	10	Hemorrhage	R’t	4	0	49	80	54	16	NA
M	44	1.64	62	Bilateral pons	36	Infarction	R’t	4	1	36	95	60	11	1 min 59 s
M	66	1.61	59	MCA	9	Infarction	L’t	5	2	43	85	43	4	NA
M	62	1.69	70	Pontine	25	Infarction	R’t	4	1	52	80	71	15	2 min 30 s
F	54	1.57	45.6	basal ganglia	54	Hemorrhage	R’t	5	0	37	80	40	11	NA
M	46	1.77	78	Subcortical region	9	Infarction	R’t	4	1	22	55	31	4	NA
M	56	1.69	65	Subcortical region	214	Hemorrhage	R’t	4	1	40	95	42	12	NA
M	53	1.65	72	Thalamus	12	Hemorrhage	L’t	4	2	60	85	64	17	4 min 4 s
M	58	1.65	69	Pons	9	Hemorrhage	L’t	4	2	26	60	45	7	NA

Abbreviations: *F* female, *M* male, *BH* body height, *BW* body weight, *m* months, *FMA-UE* upper-extremity portion of the Fugl-Meyer assessment, *BI* Barthel Index, *WMFT* Wolf Motor Function Test, *MAS* Motor Assessment Scale, *MDT* Minnesota Dexterity Test, *NA* non-assessment, *L’t* left, *R’t* right

### Measurement device and data processing

The novel and reliable bilateral hand grip measurement components included a pair of two-axis moving arms (range 0°–150°) for each upper limb, two forearm supports mounted on the moving arms, and two bow-shaped handle dynamometers [25]. Two load cells, which could detect a 980-N force and evaluate the grip force in both hands with excellent validity and reliability, were mounted onto each dynamometer [25]. All sensors were connected to a laptop computer via an NI-6008 data acquisition card (National Instruments, Austin, TX, USA). Data analysis and storage were performed using LabVIEW, 2015 edition. The sampling frequency was set at 1 kHz.

### Maximal voluntary contraction test and bilateral hand grip-force coordination control task

Before the maximal voluntary contraction (MVC) test and bilateral hand grip-force coordination control task (hereafter referred to as the “coordination control task”), each subject was asked to sit at the testing table in front of a 24-in. LCD screen. Then, each subject was asked to position their bilateral upper limbs in the testing position with the forearms supported to prevent abnormal compensatory movements of the upper extremities. The test position was defined as the wrist in the neutral position, elbow in 100°–110° of flexion, and shoulders in 40°–50° of flexion in the sagittal plane and 30°–40° of abduction in the horizontal plane.

The MVC test was executed by asking a subject to grasp the dynamometer with their maximum grip force of both hands for the healthy and stroke groups. The experimental process and data collection followed methods described in previous studies [26, 27]. After confirming the MVC values for both hands, the lower value was used to calculate the 10, 20, and 40% MVC force values to represent light, middle, and heavy target grip forces, respectively, to be used for bilateral hand grip-force coordination control tasks for each subject.

**Fig. 1:**
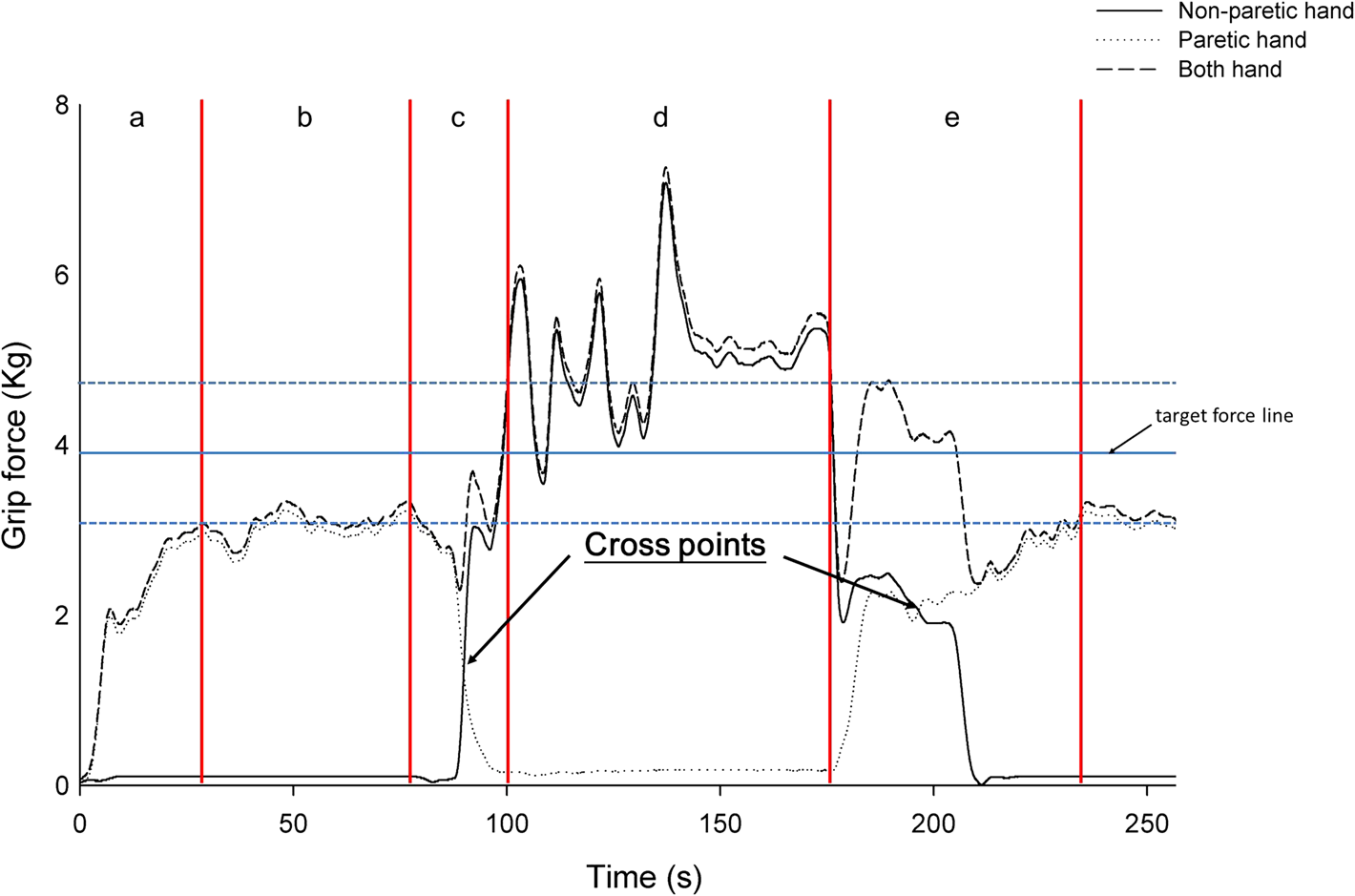
Schematic diagram of stroke subject for performing the bimanual grip force control tasks at 40% MVC target force level. **a** Grip force formation of the paretic hand: grip force was generated by the paretic hand, keep it reached the target range of application of force (3.9 ± 20% kg); (**b**) sustained grip of the paretic hand (maintain the grip force output within the target force range); (**c**) grip force was gradually released of the paretic hand and grip force formation of the non-paretic hand, keeping the resultant force of both hand was within the target force range; (**d**) sustained grip of the non-paretic hand (maintain the grip force output within the target force range); (**e**) grip force was gradually released of the non-paretic hand and grip force formation of the paretic hand, keeping the resultant force of both hand was within the target force range

The coordination control tasks were designed to mimic bilateral hand coordination control in daily activities such as grasping and manipulating an object from one hand to the other [1, 21, 23, 28]. This assessment is helpful to quantify and understand the coordination control of bilateral hand grip force [1, 28], and a recent study demonstrated that it can be used to identify age-related changes in hand coordination [21]. At the beginning of this task, each subject was asked to grasp the dynamometer using the dominant (non-paretic) or non-dominant (paretic) hand. Then, the grip force of one hand was gradually increased to the target force level. After the subject confirmed that the grip force they were exerting had reached the target force, the subject began to release the hand that was tightly grasping the dynamometer and slowly increase the grip force on the dynamometer with the other hand (Fig. 1). Condition 1 was defined as the transfer from the non-dominant to the dominant hand in a healthy subject, or from the paretic to the non-paretic hand in a stroke patient; or Condition 2 when the transfer occurred from the dominant to the non-dominant hand in a healthy subject, or from the non-paretic to the paretic hand in a stroke patient. The grip-force alternation between the two hands was collected and analyzed [21]. The summed force curve of both hand was displayed on the 24-in. LCD screen during this task. This provided visual feedback to the subjects to indicate whether the grip force of both hands had reached the target force. The instructions given to each subject were “the monitor provides the resultant force of both hands, please keep the resultant force of both hands within the target force range when you switch the grip force from one hand to the other smoothly during the task. Take your time, there is no time limit”. Therefore, each subject was performing this task at their own speed. Prior to data collection and analysis, one trial practice was performed to familiarize the subject with the task and then the trial was completed once by each subject.

### Outcome measurements for bilateral coordination control of both hands

The “alternating time” and “force applied” values for coordination with the grip force of two hands were calculated. The value of the alternating time for coordination with the grip force of two hands (denoted “alternating time” from here on) was defined as the operation time beginning from generation of the grip-force output with one hand to the cross point of bimanual grip force generation [21]. The value of the force applied for coordination with the grip force of two hands (denoted “force applied” from here on) was defined as the combined force which was exerted by both hands at the point when the hand that was applying the higher force being changed from one side to the other [21] (Fig. 1). To enable comparison of the alternating time and force applied for each subject, the data units were normalized as a percentage of the target force. The interlimb difference of alternating time and force applied from the non-dominant (non-paretic) hand to the dominant (paretic) hand and vice versa were calculated to determine the variation of coordination control of the two hands. A previous study showed that the different target force levels did not impact coordination control [21]. Therefore, we calculated the coefficient of variation (CV) values for the “alternating time” and “force applied” over the three targeted force levels from the non-dominant (non-paretic) hand to the dominant (paretic) hand, and vice versa for each subject. This revealed the overall variation in alternating time and force applied on the coordination control of individuals in this study.

### Clinical motor and functional measurements

The upper-extremity portion of the Fugl-Meyer assessment (FMA-UE) is the most commonly-used clinical assessment tool for identifying motor recovery of the paretic upper limb in stroke patients [29–31], and was employed in the present study. Functional performance evaluations included the Wolf Motor Function Test (WMFT), the Motor Assessment Scale (MAS), and the Barthel Index (BI). The WMFT is an excellent measurement tool with high reliability, and can be used to identify functional disabilities of the upper limbs in stroke patients [29, 32, 33]. The MAS is also a highly reliable tool for measuring progress in functional performance following stroke [21, 30, 34]. We also evaluated the movement time of coordination control of both hands using the turning test of the Minnesota Rate of Manipulation Test for each stroke subject, and analyzed the relationship of the results with the values of alternating time [15, 16, 18].

### Statistical analysis

Paired sample *t*-tests were applied to analyze differences in the alternating time and force applied for coordination with the grip force of two hands between the two conditions for each group and each target force level (10, 20, and 40% of the MVC). The normality of these data were evaluated using the Shapiro-Wilk Test, then the independent sample’s *t*-test was applied to analyze the differences in the alternating time and force applied of both values between groups at each target force level. The independent samples *t*-test was also used to analyze the changes in the CV values of alternating time and force applied to both hands for each coordination condition between the healthy and stroke groups. Furthermore, relationships between the values of alternating time and force applied; the scores of the FMA-UE, BI, WMFT, and MAS; and the movement time of the Minnesota Rate of Manipulation Test were analyzed using Spearman’s correlation coefficients. The alpha level of statistical significance was set at 0.05. The statistical software used was SPSS vers. 17.0 (IBM Corporation, Armonk, NY).

## Results

### Grip force performance during the bilateral coordination control task

**Fig. 2:**
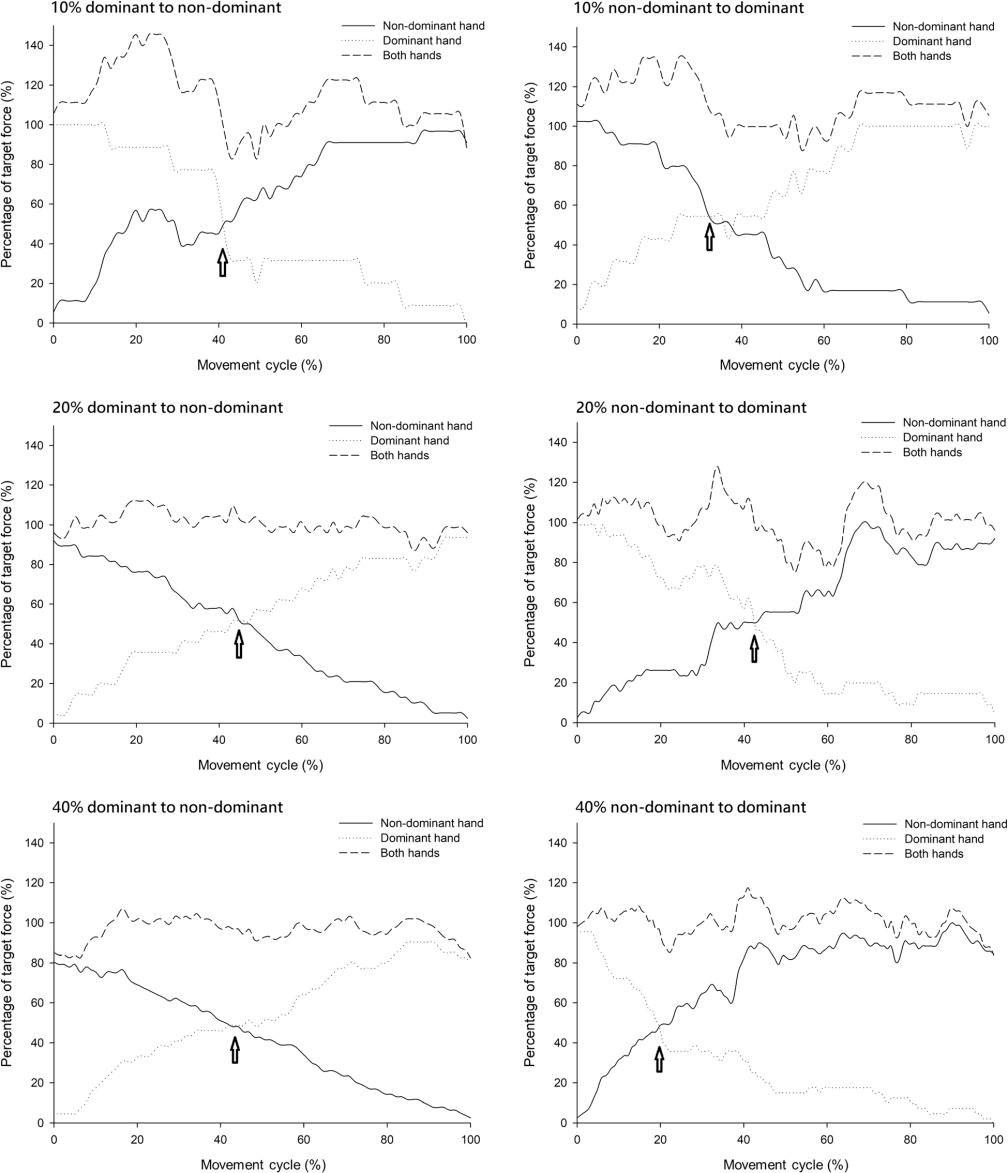
Representative plot of the grip force performance of the healthy group. The arrows indicate the cross point of the bilateral hand grip force coordination control task

**Fig. 3:**
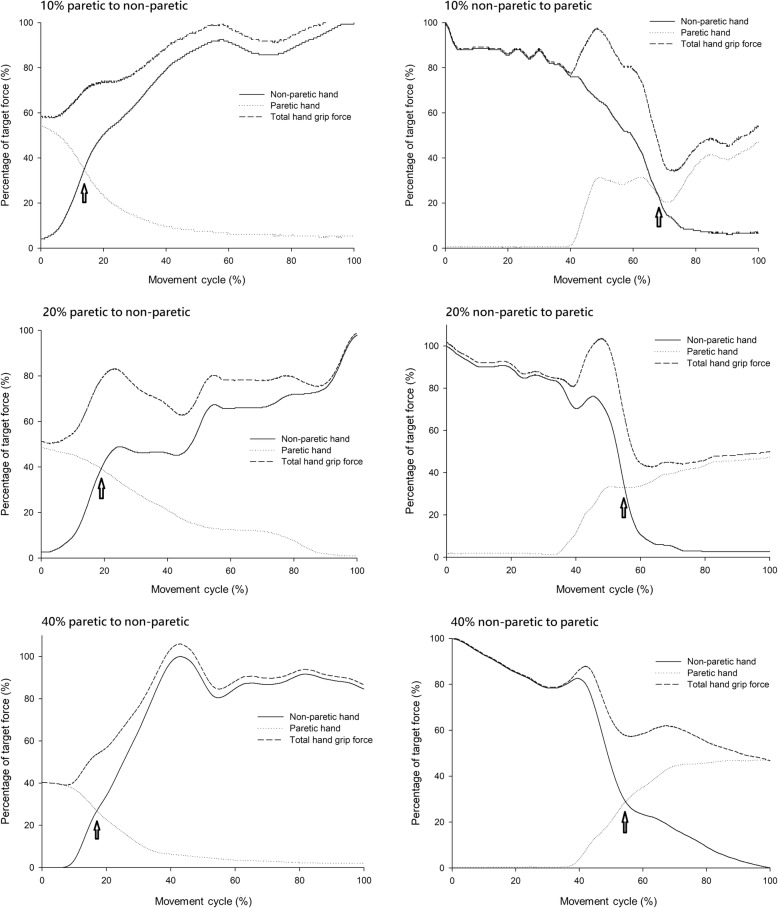
Representative plot of grip force performance of the stroke group. The arrows indicate the cross point of the bilateral hand grip force coordination control task

Figures 2 and 3 provide representative plots of grip-force performance during the coordination control task at 10, 20, and 40% MVC target force level for the healthy and stroke groups, respectively. Arrows indicate the cross point of the bilateral hand grip-force coordination control task. The resultant force of both hands did not deviate by more than 20 % at the 10 and 20% target force levels during the transition of grip force from one hand to the other in healthy subjects. This was not true for at the 40% MVC target force level (Fig. 2), where the resultant force of both hands was found to change very suddenly. The resultant force of both hands at the cross point was found to be lower in the 10, 20, and 40% target force levels during the transition of grip force from the non-paretic to the paretic hand than vice versa in the stroke group (Fig. 3). In stroke patients, longer reaction times and sudden rebounds in resultant force were detected at the beginning of the grasp for the paretic hand (Fig. 3). Additionally, compared with the non-paretic hand, we found that the starting grip forces of patients’ paretic hands were lower at the beginning of the transition of grip force from the paretic to non-paretic hand at all target force levels, although the starting grip forces did increase with increasing target force level. Stroke patients generated some grip and residual forces with the non-paretic hand when the paretic hand started to generate grip force, and the grip-force output of this hand was maintained within the target force range (Figs. 1 and 3). This phenomenon may have arisen because neuronal excitation in the ipsilateral corticospinal pathways from the affected hemisphere was induced [35].

### Stroke-related changes in bimanual grip force coordination control performance

The alternating time was shorter from the non-dominant to the dominant hand, than vice versa in the healthy group at 10% (*p* < 0.001), 20% (*p* = 0.003), and 40% (*p* = 0.001) of the MVC (Table 2). Additionally, the force generated by healthy subjects was found to be significantly higher during the transition from the non-dominant to the dominant hand, than vice versa at 40% of the MVC (*p* < 0.001) (Table 2). This indicates that bimanual grip force coordination control is faster and more powerful in the dominant hand than the non-dominant hand in healthy subjects. Furthermore, the stroke group also exhibited shorter alternating times from the paretic to the non-paretic hand than vice versa at 10% (*p* < 0.001), 20% (*p* < 0.001), and 40% (*p* = 0.004) of the MVC (Table 2), which reveal that bimanual grip force coordination control of stroke patients is significantly delayed in the paretic hand compared with the non-paretic hand. However, we found that the alternating time was shorter for stroke patients than healthy patients in Condition 2 (*p* < 0.001), which means that the bimanual grip force coordination from the non-paretic to the paretic hand in stroke patients was faster than that from the dominant to the non-dominant hand in healthy subjects. Additionally, we found that the force applied value of the healthy group was significantly larger (11.2–23.2%) than that of the stroke group at the 40% MVC target force level by (*p* < 0.05) (Table 2). The coefficient of variation (CV) values for alternating time and force applied ranged from 9.9 to 60.0% and 3.0 to 34.8%, respectively in the healthy group, and from 9.0 to 89.0% and 7.0 to 64.0%, respectively in the stroke groups. Results also showed that the CVs of the alternating time (*p* = 0.001) and force applied (*p* = 0.002) for coordination with the grip force from the non-paretic to the paretic hand in the stroke group were significantly higher than that from the dominant to the non-dominant hand in the healthy group (16.2 and 17.2%, respectively) (Table 3).

**Table 2:** Alternating time and force applied for bimanual grip force coordination tasks according to three targets of application of grip force of two hands among the healthy and stroke groups

Target force level	Condition	Outcome	Healthy group (*n* = 16)				Stroke group (*n* = 22)				Between groups	
			Mean ± SD	Difference	*t*	Sig. (2-tailed)	Mean ± SD	Difference	*t*	Sig. (2-tailed)	*t*	Sig. (2-tailed)
Total	Condition 1	Alternating time (%)	28.3 ± 8.6	18.3 ± 6.3	−11.046	0.000**	24.6 ± 8.0	23.2 ± 11.7	−5.321	0.000**	1.355	0.184
	Condition 2		59.9 ± 7.0				43.4 ± 12.8				4.692	0.000**
	Condition 1	Force applied (%)	43.7 ± 8.6	12.9 ± 5.2	−3.774	0.002*	50.6 ± 18.9	16.7 ± 15.4	.731	0.473	−1.357	0.183
	Condition 2		52.8 ± 6.0				47.3 ± 17.9				1.193	0.241
10% MVC	Condition 1	Alternating time (%)	27.5 ± 8.3	23.2 ± 15.3	−4.698	0.000**	24.6 ± 10.2	21.8 ± 15.6	−5.282	0.000**	0.944	0.352
	Condition 2		48.7 ± 17.5				44.6 ± 15.1				0.774	0.444
	Condition 1	Force applied (%)	57.7 ± 13.0	11.3 ± 8.0	0.794	0.440	60.8 ± 26.8	16.4 ± 18.3	1.094	0.286	−0.408	0.685
	Condition 2		55.0 ± 9.4				55.1 ± 23.0				−0.013	0.990
20% MVC	Condition 1	Alternating time (%)	30.2 ± 12.3	12.2 ± 6.4	−3.572	0.003*	22.0 ± 9.3	22.5 ± 16.7	−4.503	0.000**	2.347	0.025*
	Condition 2		39.5 ± 7.2				41.5 ± 16.7				−0.442	0.661
	Condition 1	Force applied (%)	59.7 ± 10.6	13.2 ± 7.9	1.457	0.166	51.8 ± 22.1	21.5 ± 24.4	0.435	0.668	1.306	0.200
	Condition 2		54.3 ± 10.2				48.8 ± 24.2				0.852	0.400
40% MVC	Condition 1	Alternating time (%)	27.1 ± 13.3	19.4 ± 9.7	−4.078	0.001*	27.3 ± 12.4	25.3 ± 14.8	−3.197	0.004*	−0.041	0.967
	Condition 2		42.7 ± 13.7				44.0 ± 18.9				−0.218	0.829
	Condition 1	Force applied (%)	62.3 ± 9.9	14.2 ± 9.9	4.634	0.000**	39.1 ± 18.0	12.3 ± 13.8	0.314	0.757	4.658	0.000**
	Condition 2		49.1 ± 6.1				37.9 ± 17.0				2.527	0.016*

Note: Condition 1 was transfer from the non-dominant to the dominant hand in a healthy subject or from the paretic to the non-paretic hand in a stroke patient; Condition 2 was transfer from the dominant to the non-dominant hand in a healthy subject or from the non-paretic to the paretic hand in a stroke patient

Note: Time was the alternating time for bimanual grip force coordination tasks indicating the transformation of grip force from both hands

Note: Force was the force applied for bimanual grip force coordination tasks indicating the transformation of grip force from both hands

* Significant difference *p* < 0.05, ** *p* < 0.001

**Table 3:** Comparison of the coefficient of variation (CV) of alternating time and force applied for bimanual grip force coordination control during two conditions among the healthy and stroke groups

Outcome	Condition	Healthy group (*n* = 16)		Stroke group (*n* = 22)		Between group	
		Mean	SD	Mean	SD	*t*	Sig. (2-tailed)
Alternating time	Condition 1	34.6%	14.5%	30.9%	16.0%	0.735	0.467
	Condition 2	15.3%	10.3%	31.5%	16.3%	−3.500	0.001*
Force applied	Condition 1	26.6%	13.6%	32.4%	18.8%	−1.042	0.305
	Condition 2	14.0%	6.1%	31.2%	19.4%	−3.417	0.002*

Note: Condition 1 was transfer from the non-dominant to the dominant hand in a healthy subject or from the paretic to the non-paretic hand in a stroke patient; Condition 2 was transfer from the dominant to the non-dominant hand in a healthy subject or from the non-paretic to the paretic hand in a stroke patient

* Significant difference *p* < 0.05

### Relationships between bimanual grip force coordination control and clinical motor and functional performances in the stroke group

The results demonstrated that the alternating time from the paretic hand to the non-paretic hand among the target force levels was significant moderately correlated with FMA-UE scores (*r* = − 0.533; *p* = 0.011), WFMT scores (*r* = − 0.450; *p* = 0.036), and BI scores (*r* = − 0.497; *p* = 0.019) (Table 4). Results also showed that the alternating time from the paretic hand to the non-paretic hand for 10% of the MVC was significantly moderately correlated with the motor performances of the FMA-UE scores (*r* = − 0.567; *p* = 0.006), WMFT scores (*r* = − 0.624; *p* = 0.002), and the motor performance of MAS scores (*r* = − 0.533; *p* = 0.011) (Table 4). These findings demonstrated that the shorter alternating time from the paretic hand to the non-paretic hand was moderately to highly correlated with motor and functional performances (increased FMA-UE, WMFT, and MAS scores) in paretic hand again. Results also demonstrated that the difference in alternating time of both hands at 20% of the MVC was significantly moderately correlated with FMA-UE (*r* = 0.512; *p* = 0.015), WMFT (*r* = 0.441; *p* = 0.040), MAS (*r* = 0.470; *p* = 0.027), and BI scores (*r* = 0.467; *p* = 0.028) (Table 4).

**Table 4:** Correlations between the alternating time and force applied for bimanual grip force coordination control, clinical motor, and functional outcome measurements (*n* = 22)

		FMA-UE		BI		WMFT		MAS	
		Pearson Correlation	Sig. (2-tailed)	Pearson Correlation	Sig. (2-tailed)	Pearson Correlation	Sig. (2-tailed)	Pearson Correlation	Sig. (2-tailed)
FMA_UE		1	–	0.554**	0.008	0.926**	0.000	0.924**	0.000
BI		0.554*	0.008	1	–	0.521*	0.013	0.503*	0.017
Wolf		0.926**	0.000	0.521*	0.013	1	–	0.902**	0.000
MAS		0.924**	0.000	0.503*	0.017	0.902**	0.000	1	–
10% MVC	Condition 1 Alternating time (%)	−0.567^*^	0.006	−0.358	0.102	−0.624^*^	0.002	−0.533^*^	0.011
	Condition 2 Alternating time (%)	0.030	0.894	0.091	0.687	0.071	0.753	−0.041	0.857
	Diff of Alternating time (%)	0.327	0.138	0.249	0.264	0.422	0.050	0.258	0.246
	Condition 1 Force applied (%)	−0.027	0.905	0.041	0.855	−0.057	0.802	0.012	0.959
	Condition 2 Force applied (%)	0.013	0.954	−0.032	0.887	−0.001	0.998	−0.154	0.494
	Diff of Force applied (%)	−0.074	0.744	0.080	0.723	−0.123	0.587	−0.016	0.942
20% MVC	Condition 1 Alternating time (%)	−0.322	0.144	−0.528^*^	0.012	−0.276	0.214	−0.147	0.515
	Condition 2 Alternating time (%)	0.360	0.099	0.306	0.165	0.311	0.159	0.345	0.116
	Diff of Alternating time (%)	0.512*	0.015	0.467*	0.028	0.441*	0.040	0.470*	0.027
	Condition 1 Force applied (%)	0.009	0.969	−0.016	0.945	−0.030	0.893	0.051	0.821
	Condition 2 Force applied (%)	−0.130	0.564	−0.206	0.357	−0.053	0.813	−0.205	0.359
	Diff of Force applied (%)	0.038	0.866	−0.062	0.784	0.055	0.809	0.072	0.750
40% MVC	Condition 1 Alternating time (%)	−0.324	0.141	−0.180	0.422	−0.241	0.280	−0.212	0.343
	Condition 2 Alternating time (%)	0.226	0.311	0.027	0.905	0.183	0.415	0.188	0.403
	Diff of Alternating time (%)	0.017	0.941	−0.066	0.772	−0.034	0.880	0.059	0.795
	Condition 1 Force applied (%)	0.031	0.892	0.111	0.622	−0.052	0.820	0.113	0.618
	Condition 2 Force applied (%)	−0.149	0.507	0.053	0.816	−0.119	0.598	−0.083	0.712
	Diff of Force applied (%)	0.015	0.948	−0.032	0.887	−0.035	0.877	0.069	0.760
Condition 1 Total Alternating time (%)		−0.533^*^	0.011	−0.450^*^	0.036	−0.497^*^	0.019	−0.393	0.070
Condition 2 Total Alternating time (%)		0.000	0.998	0.049	0.830	−0.055	0.808	0.061	0.788
Total Diff of Alternating time (%)		0.397	0.067	0.306	0.166	0.385	0.077	0.364	0.096
Condition 1 Total Force applied (%)		0.281	0.205	0.183	0.415	0.254	0.254	0.227	0.309
Condition 2 Total Force applied (%)		−0.100	0.657	−0.090	0.690	−0.062	0.784	−0.185	0.410
Total Diff of Force applied (%)		−0.005	0.984	−0.011	0.962	−0.030	0.894	0.052	0.817

Abbreviations: *FMA-UE* upper-extremity portion of the Fugl-Meyer assessment, *BI* Barthel Index, *WMFT* Wolf Motor Function Test, *MAS* Motor Assessment Scale, *MVC* maximal voluntary contraction

Note: Condition 1 was transfer from the paretic to the non-paretic hand in a stroke patient; Condition 2 was transfer from the non-paretic to the paretic hand in a stroke patient

* Significant difference *p* < 0.05, ** *p* < 0.00

Only eight stroke patients finished the turning test of the Minnesota Rate of Manipulation Test. The average movement time for all stroke patients (*n* = 8) was 228.6 s. The average movement time from the paretic to the non-paretic hand was 50.1 ± 26.4 s, and that from the non-paretic to the paretic hand was 63.8 ± 20.1 s. A significant negative correlation was observed between the movement time and the WMFT scores (*r* = − 0.805; *p* = 0.016). The alternating time value and movement time from the paretic to the non-paretic hand were highly negatively correlated (*r* = − 0.779; *p* = 0.023), as were the alternating time and movement time from the non-paretic to the paretic hand (*r* = − 0.812; *p* = 0.014).

## Discussion

### Grip-force performances during bilateral coordination control tasks are reduced in stroke patients

The resultant force of both hands did not deviate by more than 20 % at the 10 and 20% target force levels during the transition of grip force from one hand to the other in healthy subjects. This indicates that the grip force generation was very smooth, and that there was excellent stable coordination control between both hands. Compared with the healthy group, we found that the coefficient of variation (CV) values of alternating time and force applied for bimanual grip force coordination control in condition 2 in the stroke group were significantly higher by 16.2 and 17.2%, respectively (Table 3), which indicates unstable coordination control in stroke patients. Early studies have shown that the performance of coordination control tasks involving the hands decreases in elderly adults, indicated by longer reaction times of both the dominant and non-dominant hands. This results in lower resultant forces of both hands at the moment of transition of grip force from one hand to the other. However, after a few seconds’ modulation, elderly subjects can gradually and smoothly increase the grip force to return the resultant force of both hands to within the target range [21]. Functional degeneration of the neuromuscular and central nervous systems may result in decreased coordination between hands in elderly people by decreasing the spatial and temporal coordination, and therefore reducing the elderly subject’s hand dexterity [36, 37]. However, compared with the age-matched elderly group in our previous study, the stroke patients of the present study showed reduced ability to modulate the resultant force of both hands to reach the target force level when transitioning the grip force from the non-paretic to the paretic hand. There are several reasons that could result in longer reaction times of the paretic hand, lower resultant forces of both hands, and sudden rebounds and lower resultant forces that were observed in the stroke group rather than the hand preference. These include lesions of the brain, muscle weakness in the paretic hand, and reduced grip-strength control [38–42]. A longer reaction time could be induced by delayed activation from the paretic side [43], and the brain lesions of stroke patients may affect the functional performance of the ipsilateral hand [38, 39]. This is particularly likely in the case of lesions of the primary motor cortex (M1) and supplemental motor area (SMA) planning structures [40], which are key activators during bimanual arm movements [41]. Sudden rebounds of grip force of the paretic hand and resultant forces of both hands could result from muscle weakness of the paretic hand and impairment of interlimb coordination, or poor grip perception in stroke patients [44]. Additionally, recent study indicated that the grip strength of both the affected and unaffected hands are decreased [45]. The neuronal excitation of ipsilateral corticospinal pathways from the unaffected hemisphere could be induced by enhancing the activity in the non-paretic limb in patients with hemiplegia [35, 46], which could explain why we found several stroke patients unconsciously generating a lower grip force in the paretic and non-paretic hands when the other hand started to generate a grip force during the transition of grip force in both hands or maintained the grip force within the target force range. This phenomenon has been reported in previous studies that demonstrated that the muscle strength of the paretic hand increased when the non-paretic hand was exercised [47, 48].

### Differences and variations in the bimanual grip force coordination control in the healthy and stroke groups

The reduced alternating time from the paretic to the non-paretic hand at 10, 20, 40% MVC in the stroke group was somewhat unsurprising because these values may be influenced by the longer reaction time, muscle weakness, and reduced grip strength control of the paretic hand [38, 39, 42, 43, 45]. Bi and Wan also reported that the reaction times of wrist flexion and extension were longer in the paretic upper extremity than the other extremity in stroke patients [49]. However, the faster alternating time of bimanual grip force coordination from the non-paretic to the paretic hand of stroke patients compared with that from the dominant to non-dominant hand of healthy subjects was surprising, as we would expect an increase in the reaction time of stroke patients. This phenomenon might be a compensatory movement because the monitor provides the resultant force of both hands, and the patients found that the paretic hand could not generate and modulate sufficient grip force to match the decreasing grip force of the non-paretic hand during coordination control of both hands. This caused the stroke patients to generate grip force with the paretic hand as fast as they could during the coordination control of both hands [38, 39, 42, 43, 45]. Sudden rebound is then induced when the grip force is switched from the non-paretic to the paretic hand, which is the most obvious evidence of this phenomenon. The lower force generation for coordination with the grip force in the stroke group at the 40% MVC target force level may suggest that if we need to differentiate the stoke-related changes in grip force generation during the coordination control task, the heavy target grip forces could be required. A grip-force task with increased output measurements may be helpful to identify the lower grip force generation during coordination control of both hands in the stroke group.

The high CVs of the alternating time and force applied from the non-paretic to the paretic hand in the stroke group and from the dominant to the non-dominant hand in the healthy group compared with vice versa between both groups indicate increased instability in the stroke group. This may be due to central neuron lesions, which result in muscle weakness and reduced grip-strength control in the paretic limb [38–42]. This leads to spatially and temporally uncoordinated movements [21, 24] and less smooth movement tracking or a greater range of deviation [20]. In contrast, a previous study revealed the CVs of alternating time and force applied for coordination with the grip force of both hands to be 29.7–59.1% and 57.5–79.9% in elderly subjects, respectively [21]. These values are higher than those of the stroke group in the present study. The previous study required each subject to perform the bilateral coordination tasks three times in 30 s, whereas we put no time limit on the present study because we predicted stroke patients to need more time to accomplish the task. While the previous study found no significant difference in the alternating time between hands for all three target MVC levels in healthy subjects [21], we detected a shorter alternating time from the non-dominant to the dominant hand than vice versa in the healthy group at all target force levels in this study. Therefore, these different findings may result from the time constraints and indirectly suggest that if we need to differentiate the grip-force control during the bilateral hand grip force coordination control task, time constraints may not be required and placing no limitations on time may actually be beneficial. The previous study also showed that the differences in alternating times of both hands in young and elderly adults were 12.4–20% and 12–24.7%, respectively, at three target force levels [21]. Similarly, we found the difference in alternating times for both hands in the stroke group (23.2 ± 11.7%; range 21.8–22.5%) to be higher than the healthy group (18.3 ± 6.3%; range 12.2–23.2%). The differences in alternating time of both hands in the stroke group of the present study were large, and were in fact greater than those of elderly people [21].

### The relationship between bimanual grip force coordination control and clinical motor and functional performances in the stroke group

The positive correlation of alternating time from the paretic to the non-paretic hand with FMA-UE, WFMT, and BI scores suggests that movement times from the paretic to the non-paretic hand are associated with motor and functional performances in the paretic upper extremity. This was a surprising result because we expected these performances would be associated with a shorter movement time from the non-paretic to the paretic hand. However, early studies have demonstrated that neuronal excitation of ipsilateral corticospinal pathways in the unaffected hemisphere can be induced by activity in the paretic limb [35, 46]. This suggests that the faster movement time that we observed for the non-paretic hand could be due to neuronal excitation of the damaged hemisphere, which led to better motor and functional performance in the paretic upper extremity [38, 50]. This is supported by our finding that shorter alternating times were moderately associated with enhanced motor and functional performances.

The movement time for turning test of the Minnesota Rate of Manipulation Test indicated that increased dexterity in the paretic hand was related to improved functional performance of the paretic limb in stroke patients. Additionally, the negative correlation of the movement time with alternating time value indicated that the abnormal shortening of the alternating time in stroke patients may have been induced by the longer reaction times, muscle weakness, and reduced grip strength control of the paretic hand [38–43]. These factors affect the dexterity of the hand and increase the movement time. These observations can be explained by our hypothesis of compensation for coordination control, which was described earlier.

### Study contributions and limitations

This study was the first time that we evaluated bilateral hand grip force coordination control in stroke patients using an assessment system with two embedded dynamometers and carried out quantitative analysis of the coordination control between the hands. To summarize, the first major finding was the observation that muscle weakness and longer reaction times of the paretic hand of stroke patients induced a sudden rebound in grip force, resulting in sudden changes in the resultant force of both hands when the paretic hand started generating the grip force during bilateral coordination control. The second major finding was that stroke patients generated detectable grip and residual forces in the non-paretic hand when the paretic hand started to grasp the dynamometer and while the grip-force output of this hand was maintained within the target force range. The third finding was the compensative movement which resulted in shorter alternating times and higher CVs of the alternating time and force applied from the non-paretic to the paretic hand compared with vice versa. The stroke-related changes in alternating time and force applied for coordination with the grip force of both hands are not often discussed in the literature because most therapists use clinical motor and functional assessment forms to evaluate the motor and functional performance of the paretic upper extremity, and so the coordination control of both hands in stroke patients is rarely discussed [21, 29–34]. Additionally, this shorter alternating time could be used to follow and identify the effects of rehabilitation on the motor and functional recovery progress of the paretic hand in stroke patients. Finally, the alternating time was found to be strongly negatively correlated with the movement time for turning test of the Minnesota Rate of Manipulation Test, which indicates that the abnormal shortening of the alternating time from the non-paretic to the paretic hand is induced by muscle weakness, reduced grip-strength control, and longer reaction times in the paretic hand [38–43], and is significantly correlated with reduced dexterity of this hand. According to these findings, we suggest that any rehabilitation programs should focus not only on muscle strength training for the paretic limb, but also on reaction time and muscle strength control training. Additionally, the training should provide feedback, especially during coordination control of both hands, as this would enable the patient to improve the reaction time and muscle strength of the paretic limb.

The present study had some limitations. The variation in Brunnstrom stage was relatively large; therefore, further studies may be required to specifically assess whether the different Brunnstrom stages are associated with different outcomes of coordination control in both hands. Another limitation was that the age and sex status were unmatched between the healthy and stroke groups. The average age of the stroke group was 53.7 years, with two patients over 65 y/o; therefore, the effect of aging on coordination control may have affected the results of this study. Finally, the sample size was small, and larger samples may be necessary to draw firm conclusions. Additionally, these findings provide some data on discriminative and concurrent criterion validity, which is necessity of further studies to establish the psychometric properties (reliability, responsiveness) of this method.

## Conclusions

The results indicate that stroke results in unstable and poor bilateral hand coordination control during the transfer of handgrip strength to the paretic side. This study demonstrated that a shorter alternating time from the paretic hand to the non-paretic hand was positively correlated with better motor and functional performance in stroke patients.

## Data Availability

Please contact the corresponding author for data requests.

## Abbreviations

BI: Barthel Index
FMA-UE: Upper-extremity portion of the Fugl-Meyer Assessment
MAS: Motor Assessment Scale
MVC: Maximal voluntary contraction
WMFT: Wolf Motor Function Test
